# Lateral palatal foramina are ubiquitous across fossil whales and shared with hippos

**DOI:** 10.1098/rsos.261050

**Published:** 2026-10-21

**Authors:** Nicholas D. Pyenson, Mark D. Uhen, Carlos Mauricio Peredo

**Affiliations:** ^1^ Department of Paleobiology, National Museum of Natural History, Smithsonian Institution, Washington, DC, USA; ^2^ J. Shukla Department of Atmospheric, Oceanic, and Earth Sciences, George Mason University, Fairfax, VA, USA; ^3^ Vivitec AI, Crestview Hills, KY, USA

**Keywords:** baleen, filter-feeding, Cetacea, marine mammals, teeth

## Abstract

Lateral palatal foramina have been proposed as osteological correlates for baleen in fossil mysticetes. A recent study reported significantly elevated foramina counts in extant mysticetes relative to terrestrial artiodactyls (Ekdale *et al*. 2024 *Sci. Rep.* 14, 10174 (doi:10.1038/s41598-024-60673-8)). However, that analysis drew conclusions about fossil taxa despite not including any fossil taxa in its analysis. Here, we merge the datasets of Peredo *et al.* (Peredo *et al.* 2022 *Sci. Rep.* 12, 11448 (doi:10.1038/s41598-022-15684-8)) and Ekdale *et al.* (Ekdale *et al*. 2024 *Sci. Rep.* 14, 10174 (doi:10.1038/s41598-024-60673-8)) and add palatal foramina counts for eight stem mysticetes, producing a combined dataset of 195 specimens. Stem mysticetes are statistically indistinguishable from terrestrial artiodactyls in the number of palatal foramina (stem mysticete median = 4; artiodactyl median = 3) and significantly different from extant mysticetes (median = 46.5). Finer taxonomic analysis reveals that the elevated mysticete signal is driven by Balaenopteroidea. Balaenidae and Neobalaenidae, despite bearing baleen, do not exhibit elevated counts. Moreover, the elevated counts in Balaenopteroidea are a condition shared with Hippopotamidae, the closest living relatives of cetaceans. These results indicate that palatal foramina counts probably reflect the degree of soft tissue vascularization rather than the presence of baleen. Lateral palatal foramina counts cannot be used to infer baleen in fossil whales, and phylogenetic bracketing remains the most defensible framework for inferring baleen in fossil cetaceans.

## 1. Introduction

Among mammals, baleen whales (mysticetes) uniquely evolved filter feeding. Unlike their terrestrial ancestors, which used a tribosphenic molar for mastication, living mysticetes have no teeth as adults [1–4]. Instead, mysticetes feed using specialized keratinous plates, called baleen, to filter feed large volumes of prey-laden water in bulk [5–8]. Baleen is a novel integumentary structure that coincides with the diversification of crown Mysticeti [9,10] and was potentially an exaptation that facilitated gigantism in whales [11]. Thus, baleen is a morphological transformation linked with macroevolutionary changes akin to the scales-to-feathers transformation in archosaurs [12,13], fins to limbs in tetrapods [14,15] and the origin of the turtle shell [16,17].

Despite its importance for understanding the broader macroevolutionary patterns in whales, the timing and mechanisms driving the origin of baleen remain obscure [4,18]. First, baleen is rare in the fossil record, with only a few occurrences linking fossilized baleen with morphologically diagnostic specimens [19–21]. The oldest reported record of fossil baleen (approx. 10 Ma; [19–22]; see [23] for ages) is less than a third the age of the oldest stem mysticetes (approx. 36 Ma; [24,25]). Thus, there is an approximately 26 million year gap between the likely origin of stem mysticetes and the oldest direct evidence for fossil baleen. Until this gap is closed, the presence or absence of baleen in fossil whales must be inferred from osteological correlates, phylogenetic bracketing, or the occurrence of older fossilized baleen [26].

Morphological and molecular evidence overwhelmingly support a single origin of baleen in whales [27–29]. Therefore, phylogenetic bracketing [30] supports the inference of baleen within crown Mysticeti. Previous authors have sought to infer baleen outside of the crown using lateral palatal foramina as osteological correlates for baleen [31,32]. These foramina are small bony openings in the palate which, in extant baleen whales, deliver vascular and/or nervous tissue to the gingiva ( figure 1). This soft tissue, called the Zwischensubstanz because it is ‘between substances’ [34], supports the base of the baleen plates [32]. However, other authors have called into question the inference of baleen from lateral palatal foramina [4,19,26,35].

Peredo *et al.* [26] presented data showing that lateral palatal foramina are present in the palates of terrestrial artiodactyls ( figure 2) and whales without baleen, including toothed whales (odontocetes) and stem cetaceans (or archaeocetes). Ekdale *et al.* [36] replicated the analysis by Peredo *et al.* [26] and found evidence that the foramina in extant mysticetes may be superlative in size and number. They reported significant differences in the total number of palatal foramina between extant mysticetes and terrestrial artiodactyls. On this basis, Ekdale *et al.* [36] concluded that the presence of palatal foramina is sufficient for inferring the presence of baleen in extinct mysticetes. We note that Peredo *et al.* [26] was subsequently retracted by the journal despite dissent of all its authors.

Taking a step back, the disagreement between the findings by Peredo *et al.* [26] and Ekdale *et al.* [36] hinges on replicating palatal foramina counts. Replication is a vital part of the scientific process that establishes the credibility of scientific claims [37] because reproducing results confronting existing hypotheses builds towards accepted paradigms. Nosek & Errington [37] proposed that replication studies adopt a key criterion: they should bear on the same question as the original study by using novel data, methods, and/or variables to expand the dialogue. Thus, the discrepancy in counts between Peredo *et al.* [26] and Ekdale *et al.* [36] is unsurprising: identifying and classifying palatal foramina is subjective in practice, and these two studies used different criteria and methodologies.

However, several interpretations advanced by Ekdale *et al.* [36] make cross-comparisons challenging. For example, they choose to consider only palatal foramina on the maxilla, while excluding those on the premaxilla or palatine from their statistical analyses. Moreover, they did not consider foramina near the alveolar margins or in the inter-alveolar septae. Crucially, palatal foramina appear in these regions for stem mysticetes, such as *Aetiocetus weltoni*, and their presence in toothed mysticetes suggests exaptation of dental development to support baleen. Finally, the conclusions drawn by Ekdale *et al.* [36] omitted fossil taxa from their analyses ([36, figure 3] and supplementary material, table S2). Thus, the data compiled by Ekdale *et al.* [36] cannot directly bear on the question of baleen in fossil mysticetes.

To move this latter question forward, we review here the discrepancy in palatal foramina counts by previous authors [26,36] and merge the disparate datasets into a new and comprehensive dataset of palatal foramina counts in living and fossil whales, along with terrestrial artiodactyls. Next, we add palatal foramina counts for eight stem mysticetes. Finally, we re-analyse the dataset and expand on previous efforts by directly testing for differences between stem mysticetes and other taxonomic groups. We directly test the hypotheses that: (1) extant mysticetes have a superlative number of palatal foramina; and (2) the palatal foramina of stem mysticetes imply the presence of baleen, as asserted by Ekdale *et al.* [36].

## 2. Material and methods

The Peredo *et al.* [26] dataset contains 96 data points spanning 16 species of modern and fossil whales and 61 species of extant terrestrial artiodactyls (supplementary material, table S1). The Ekdale *et al.* [36] dataset contains 100 data points spanning 39 species of modern whales and 61 species of extant terrestrial artiodactyls (supplementary material, table S2). The Ekdale *et al.* [36] dataset contains no fossil taxa. In total, only eight fossil taxa were included in both datasets and are reported, with their respective counts, in supplementary material, table S3. Together, these disparate datasets produce a combined dataset with counts for 188 taxa spanning 46 modern and fossil whales and 88 species of extant terrestrial artiodactyls (supplementary material, table S1).

To this combined dataset, we added palatal foramina counts for seven stem mysticete taxa not included in either previous study: *Maiabalaena nesbittae*, *Llanocetus denticrenatus*, *Coronodon havensteini*, *A. weltoni*, *Fucaia goedertorum*, *Niparajacetus palmadentis* and *Mystacodon selenensis* (table 1). Counts for these taxa were obtained from published photographs and descriptions in the primary literature. Additionally, *Aetiocetus cotylalveus*, which had been classified under Mysticeti in the Peredo *et al.* [26] dataset, was reclassified as stem Mysticeti and is reported alongside the other stem mysticetes in table 1. The final combined dataset thus contains 195 data points.

**Table 1.** List of specimens observed and the total number of palatal foramina reported for fossil mysticetes added to the analysis in this study. See electronic supplementary material for tabulated data from Peredo *et al.* [26] and Ekdale *et al.* [36].

taxon	group	Specimen number	palatal foramina count
*Aetiocetus cotylalveus*	stem Mysticeti	USNM PAL 25 210	1
*Maiabalaena nesbittae*	stem Mysticeti	USNM PAL 314 627	6
*Llanocetus denticrenatus*	stem Mysticeti	USNM PAL 183 022	5
*Coronodon havensteini*	stem Mysticeti	CCNHM 108	9
*Aetiocetus weltoni*	stem Mysticeti	UCMP 122 900	8
*Fucaia goedertorum*	stem Mysticeti	LACM 131 146	3
*Niparajacetus palmadentis*	stem Mysticeti	MUEcSj 524.123	2
*Mystacodon selenensis*	stem Mysticeti	MUSM 1917	3

*Institutional Abbreviations:* AMNH, American Museum of Natural History, New York, NY, USA; CCNHM, Mace Brown Museum of Natural History, College of Charleston, Charleston, SC, USA; LACM, Natural History Museum of Los Angeles County, Los Angeles, CA, USA; MUEcSj, Museo de Historia Natural, Universidad Autonoma de Baja California Sur, La Paz, Baja California Sur, Mexico; MUSM, Museo de Historia Natural, Universidad Nacional Mayor de San Marcos, Lima, Peru; UCMP, University of California Museum of Paleontology, Berkeley, CA, USA; UMMZ, University of Michigan Museum of Zoology, Ann Arbor, MI, USA; USNM, Division of Mammals, Department of Vertebrate Zoology, National Museum of Natural History, Smithsonian Institution, Washington, DC, USA; USNM PAL, Department of Paleobiology, National Museum of Natural History, Smithsonian Institution, Washington, DC, USA.

### 2.1. Disparate methods for counting lateral palatal foramina

Identifying and classifying palatal foramina in mammals is surprisingly subjective because other types of bony openings can be present. For example, damage to the bone from post-mortem (or even taphonomic) processes can result in openings that may superficially resemble foramina. Additionally, it is common for the trabecular bone of the alveolar region in mammals to be particularly porous, resulting in many bony openings that are only superficial [38–40]. Therefore, it is important to only consider true foramina that have confluent internal canals connecting to proximal canals housing nervous and vascular tissue (e.g. superior alveolar canal).

Given these challenges, Peredo *et al.* [26] sought to categorize the bony openings into major and minor palatal foramina. The authors defined major foramina as those exceeding 1 mm in diameter. Despite this lower limit, major foramina were generally quite large, appeared in isolation on the palate, and were occasionally associated with an elongated sulcus. Conversely, minor foramina (those less than 1 mm in diameter) often appeared in clusters of five or more small bony openings that lacked sulci. It is well established that the distal termination of the superior alveolar nerve sometimes branches into small nerve bundles [38–40]. Thus, Peredo *et al.* [26] included clusters of minor foramina in addition to their counts of the major foramina.

In contrast, Ekdale *et al.* [36] were primarily concerned with differentiating the palatal foramina in the maxilla from those in the palatine and premaxilla. Additionally, they did not consider the minor palatal foramina to be informative for the research question, and counted only foramina greater than 1 mm in diameter—what Peredo *et al.* [26] would call major palatal foramina. Ekdale *et al.* [36] reported the number of palatal foramina found in each bone, as well a total value for each specimen (supplementary material, table S2).

This discrepancy in methodology reflects subjective decision-making about a complex and nuanced morphology, which enriches the question at hand by broadening its scope [37]. Here, we consolidate the two datasets into a single, comprehensive record of palatal foramina in whales and artiodactyls (supplementary material, table S1). To maintain consistency of the records across the two previous studies, we focused only on values with unambiguously matching definitions. Thus, our combined dataset (supplementary material, table S1) includes the major palatal foramina, but not the minor clusters of foramina, from Peredo *et al.* [26]. Moreover, we use the total values for each specimen reported by Ekdale *et al.* [36], as Peredo *et al.* [26] did not conduct counts for the premaxillae, maxillae and palatines separately.

This new approach ensures that the combined dataset includes only palatal foramina greater than 1 mm in diameter, regardless of which bone they are present in. For the eight specimens that appeared in both studies, we deferred to the counts reported by Ekdale *et al.* [36]. The extent of this discrepancy is illustrated by *Bubalus bubalis* (UMMZ 156 568), for which Peredo *et al.* [26] reported seven major palatal foramina whereas Ekdale *et al.* [36] reported zero (figure 2). This approach has the added benefit of addressing one key critique made by Ekdale *et al.* [36], who argued that Peredo *et al.* [26] over-inflated the number of palatal foramina in terrestrial artiodactyls and undercounted the number of foramina in extant mysticetes. The methodological approach used here employs conservative counts from the Peredo *et al.* [26] dataset and higher counts from the Ekdale *et al.* [36] dataset.

**Figure 1.**
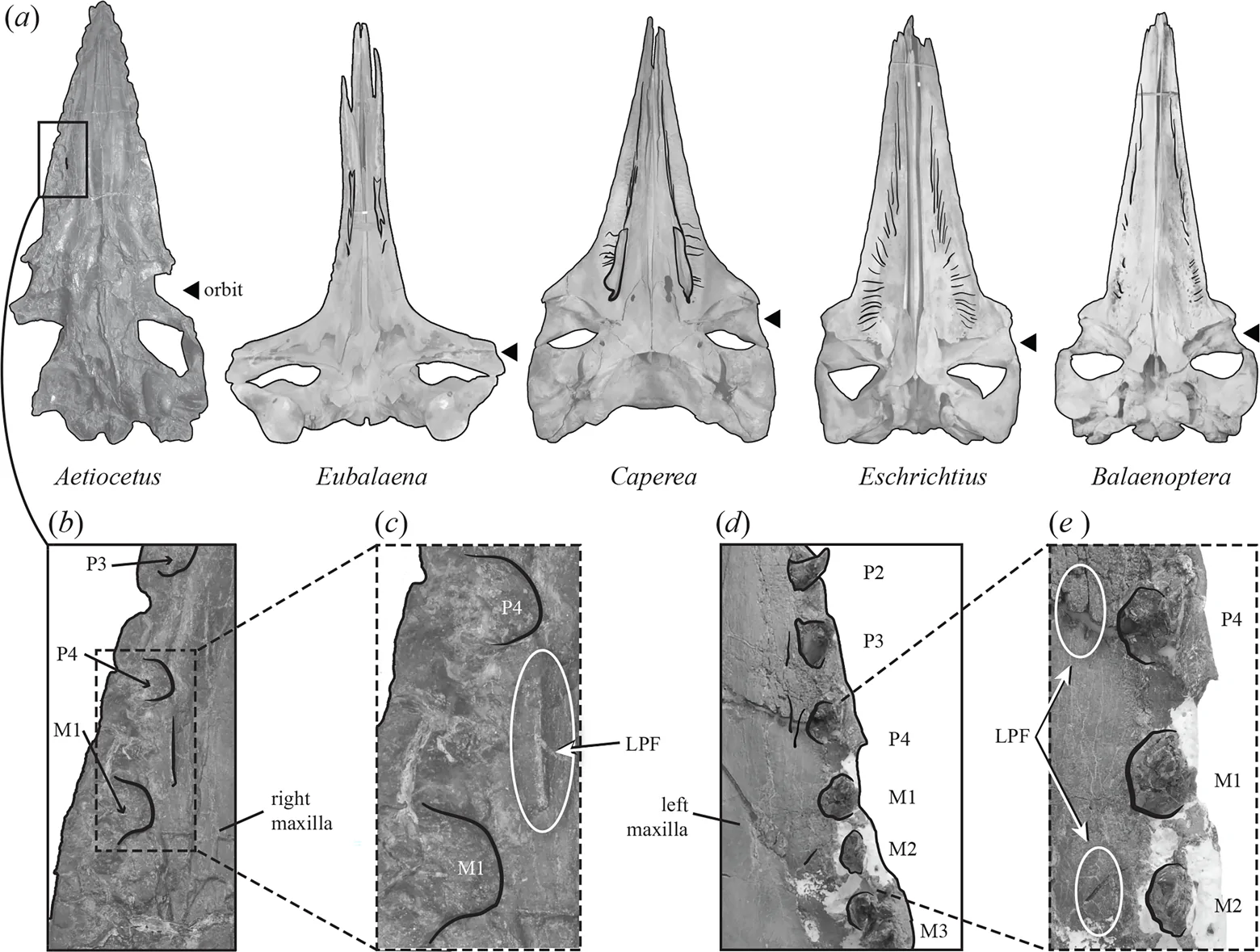
Palatal morphology of fossil and extant Mysticeti. (*a*) From left to right, the palate of *Aetiocetus cotylalveus* (USNM PAL 215 210), *Eubalaena glacialis* (USNM 301 637), *Caperea marginata* (USNM 550 146), *Eschrichtius robustus* (USNM 364 973) and *Balaenoptera borealis* (USNM 593 415), all scaled to the same condylobasal length. Black outlines highlight palatal sulci. (*b,c*) Magnified view of the palate of *Aetiocetus cotylalveus* highlighting the single possible palatal sulcus. (*d,e*) Magnified view of the palate of *Aetiocetus weltoni* highlighting four possible palatal sulci. M, upper molar; P, upper premolar; after Deméré and Berta [33]. Modified from Peredo *et al.* [4].

### 2.2. Taxonomic groupings

We conducted statistical analyses at two levels of taxonomic resolution. First, we assigned all taxa to one of five high-level groups: Artiodactyla, stem Cetacea, stem Mysticeti, Mysticeti (crown group) and Odontoceti. Second, to test for finer-scale variation within these groups, we subdivided Artiodactyla into nine families (Antilocapridae, Bovidae, Camelidae, Cervidae, Giraffidae, Hippopotamidae, Suidae, Tayassuidae and Tragulidae) and subdivided extant Mysticeti into Balaenidae & Neobalaenidae versus Balaenopteroidea (Balaenopteridae + Eschrichtiidae). Stem Cetacea, Odontoceti and stem Mysticeti were retained as single groups in this finer analysis.

### 2.3. Statistical analyses

We conducted all statistical tests in R (version 4.4.1) using RStudio (version 2024.04.2), with the packages dplyr, tidyverse, dunn.test and multcompView. Shapiro–Wilk tests were used to assess normality within each group. Because several groups significantly departed from normality (notably Artiodactyla, W = 0.26, *p* < 0.001), we used non-parametric tests as the primary statistical framework. We performed Kruskal–Wallis rank sum tests to assess overall differences across taxonomic groups, followed by Dunn's test with Bonferroni correction for pairwise post hoc comparisons. For completeness, we also conducted one-way ANOVAs with Tukey's honest significant difference (HSD) post hoc tests [41]; the parametric and non-parametric approaches yielded identical conclusions in all cases. Compact letter displays were generated using the multcompView package to summarize significance groupings. Both sets of analyses were performed at the five-group level and at the finer taxonomic subgroup level described above.

## 3. Results

### 3.1. Discrepancies between previous studies

Peredo *et al.* [26] and Ekdale *et al.* [36] reached opposing conclusions from their respective datasets (figures 3, 4 and 5). Using only major palatal foramina (more than 1 mm diameter), Peredo *et al.* [26] found no significant differences across Artiodactyla, stem Cetacea, Mysticeti and Odontoceti (one-way ANOVA, *p* > 0.05; figure 4*b*). In contrast, Ekdale *et al.* [36] reported highly significant differences across their three sampled groups (Artiodactyla, Mysticeti and Odontoceti), driven by elevated counts in extant Mysticeti (figure 4*a*). Notably, Ekdale *et al.* [36] did not sample any stem cetaceans nor stem mysticetes (figure 4*a*).

**Figure 2.**
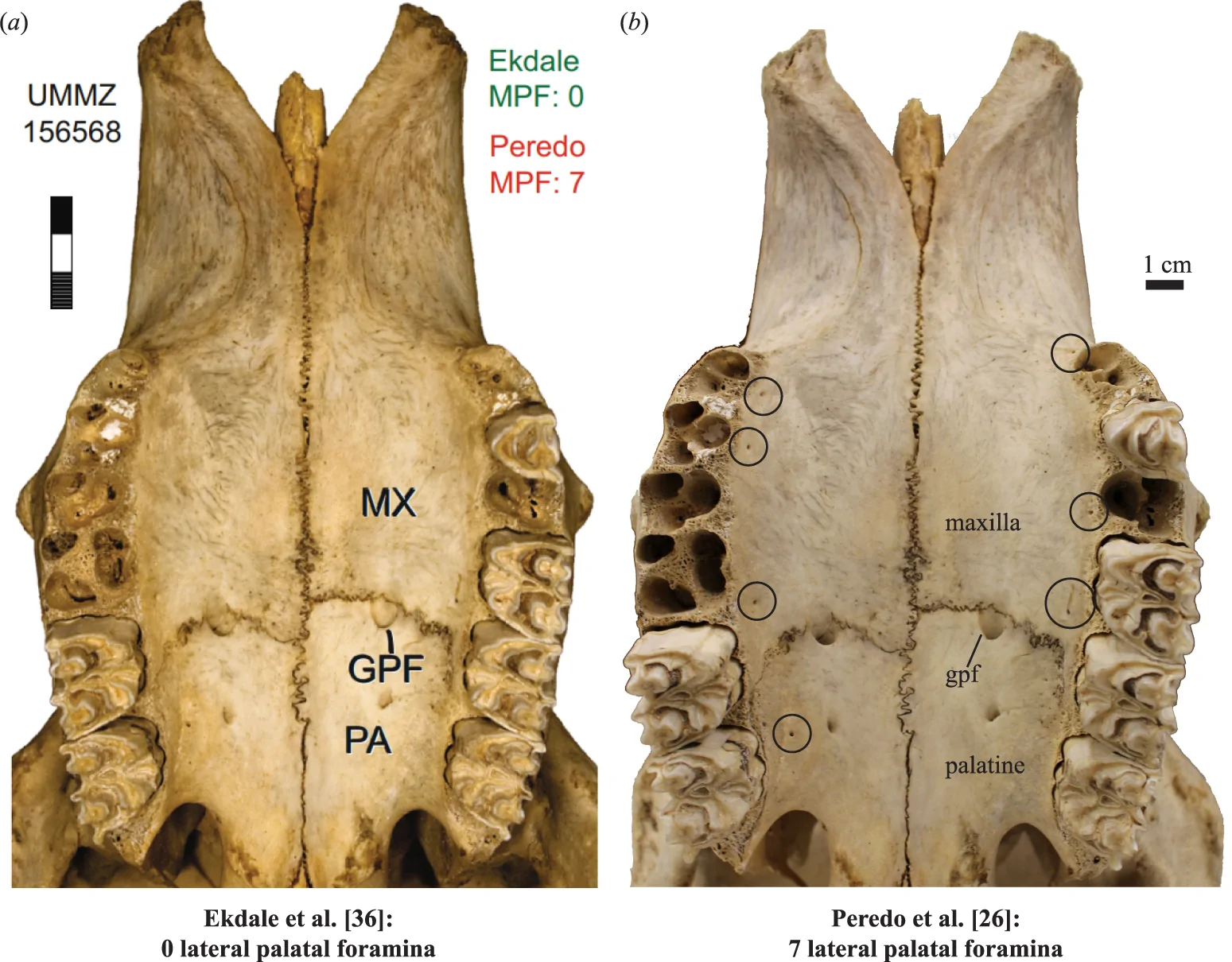
Ventral view of the palate of *Bubalus bubalis* (UMMZ 156 568), illustrating discrepancies in palatal foramina identification between studies. (*a*) Ekdale *et al.* [36] identified zero major palatal foramina on the maxilla of the same specimen, excluding all foramina identified by Peredo *et al.* [26] as belonging to the palatine canal system rather than the superior alveolar canal. (*b*) Peredo *et al.* [26] identified seven major palatal foramina (circled) on the maxilla and palatine, including foramina near the alveolar margins. Abbreviations: gpf/GPF, greater palatine foramen; MX, maxilla; PA, palatine.

The eight specimens common to both datasets reveal the degree to which palatal foramina counts are sensitive to methodological criteria (supplementary material, table S3). For example, counts for *Eschrichtius robustus* (USNM 364 973) differed by an order of magnitude between studies (Peredo *et al.* [26]: 5 major palatal foramina; Ekdale *et al.* [36]: 69 total). Conversely, Ekdale *et al.* [36] reported lower counts than Peredo *et al.* [26] for several artiodactyl specimens, including *Aepyceros melampus* (UMMZ 124 571; 2 versus 8) and *Antilocapra americana* (UMMZ 65 502; 2 versus 10). These discrepancies reflect the inherent subjectivity in identifying palatal foramina, rather than errors by either research team.

### 3.2. Addition of stem mysticetes

Neither Peredo *et al.* [26] nor Ekdale *et al.* [36] tested stem mysticetes as a distinct group. Peredo *et al.* [26] included a single stem mysticete, *A. cotylalveus* (USNM PAL 25 210; 1 foramen), among Mysticeti. Conversely, Ekdale *et al.* [36] included no fossil taxa. To address this gap, we added palatal foramina counts for eight stem mysticetes (table 1): *A. cotylalveus* (USNM PAL 25 210; 1 foramen), *A. weltoni* (UCMP 122 900; 8), *C. havensteini* (CCNHM 108; 9), *M. nesbittae* (USNM PAL 314 627; 6), *L. denticrenatus* (USNM PAL 183 022; 5), *F. goedertorum* (LACM 131 146; 3), *M. selenensis* (MUSM 1917; 3) and *N. palmadentis* (MUEcSj 524.123; 2).

**Figure 3.**
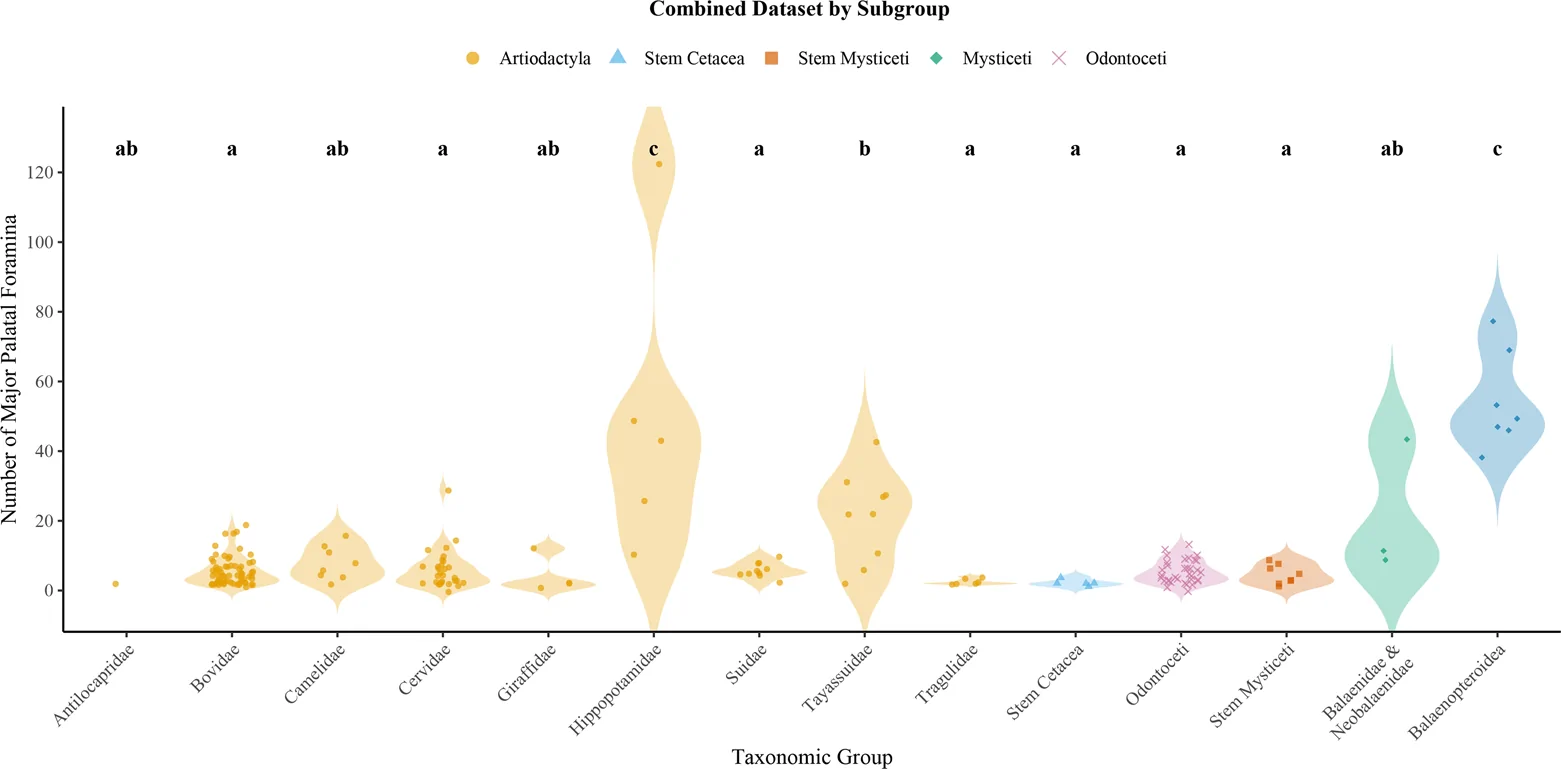
Violin plot of major palatal foramina counts in the combined dataset, with artiodactyl and mysticete taxa broken into finer taxonomic subgroups. Letters above each group indicate Tukey's HSD significance groupings. Balaenopteroidea (group ‘c’) is the primary driver of the elevated mysticete signal, while Balaenidae & Neobalaenidae (group ‘ab’) are not significantly different from most artiodactyl families. Notably, Hippopotamidae—the closest living relatives of Cetacea—also falls in group ‘c’, clustering with rorquals rather than with other artiodactyl families. Stem Mysticeti (group ‘a’) remains indistinguishable from the majority of artiodactyl families.

When these stem mysticetes are added to the Peredo *et al.* [26] dataset, they cluster with Artiodactyla, stem Cetacea and Odontoceti at the base of the distribution, well below the extant Mysticeti (figure 4*d*). This same pattern is true when stem mysticetes are added to the Ekdale *et al.* [36] dataset (figure 4*c*). In both cases, stem mysticetes are visually and statistically indistinguishable from non-mysticete groups, regardless of the applied counting methodology.

### 3.3. Combined dataset: five-group analysis

The combined dataset contains 195 data points across five groups: Artiodactyla (*n* = 138; median = 3, mean = 4.9), stem Cetacea (*n* = 5; median = 1, mean = 3.8), stem Mysticeti (*n* = 8; median = 4, mean = 4.6), Mysticeti (*n* = 10; median = 46.5, mean = 44.2) and Odontoceti (*n* = 34; median = 5, mean = 5.3). A Kruskal–Wallis test revealed highly significant differences among groups (chi-squared = 35.77, d.f. = 4, *p* = 3.23 × 10^−7^). A one-way ANOVA corroborated this result (F = 33.78, d.f. = 4, *p* < 2 × 10^−16^).

Dunn's post hoc test with Bonferroni correction identified Mysticeti as significantly different from all four other groups: Artiodactyla (*p* < 0.001), stem Cetacea (*p* < 0.001), stem Mysticeti (*p* = 0.012) and Odontoceti (*p* = 0.002). No other pairwise comparisons were significant (all *p* > 0.05). Tukey's HSD yielded identical groupings. These results are summarized by compact letter display in figure 5, where Mysticeti is the sole member of group ‘b’ and all other groups share the designation ‘a.’

**Figure 4.**
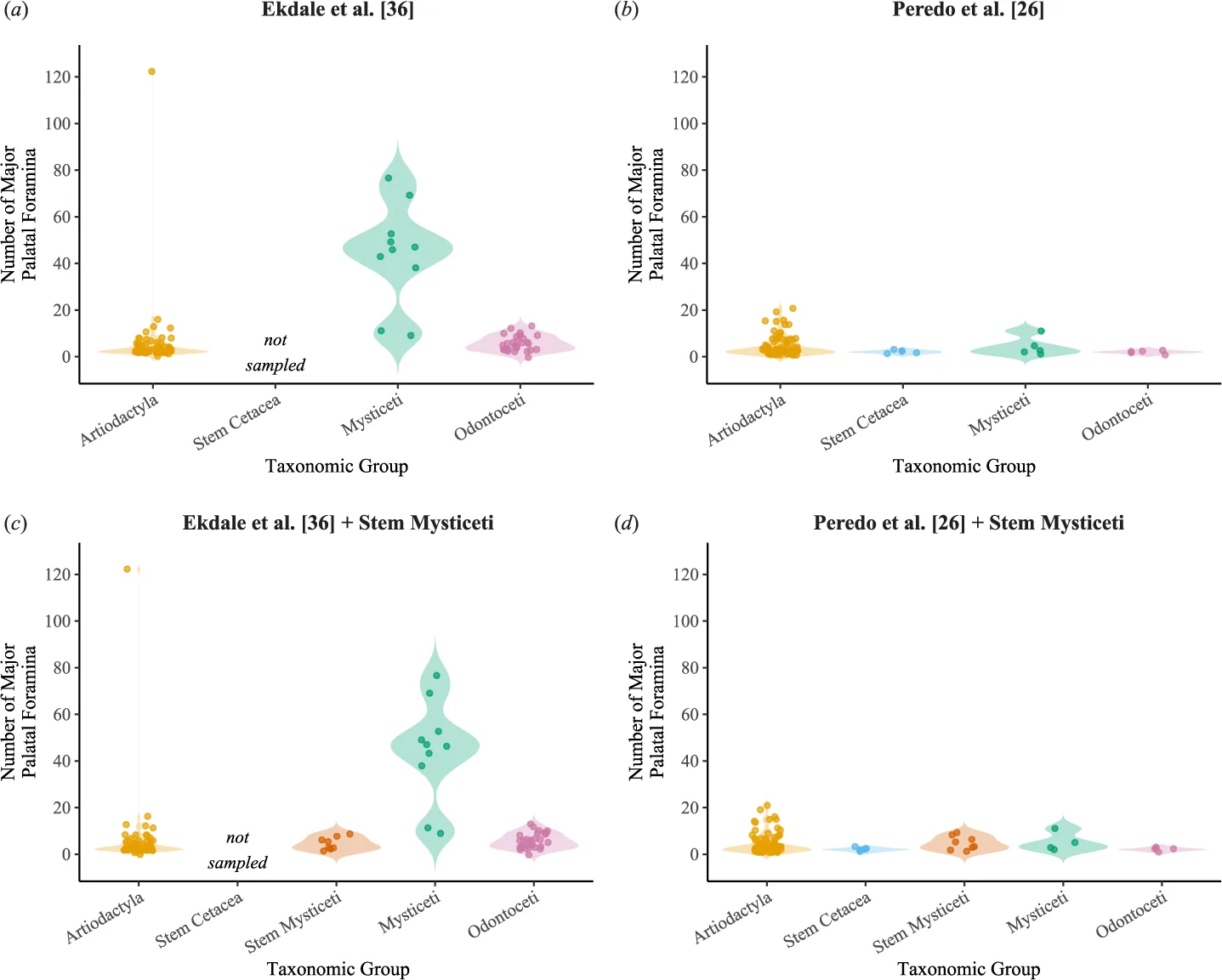
Violin plots comparing the number of major palatal foramina (more than 1 mm diameter) across taxonomic groups as reported by each study, before and after the addition of stem mysticete taxa. (*a*) Ekdale *et al.* [36] data as published, using conservative (‘Major’) counts. (*b*) Peredo *et al.* [26] data as published; note that stem cetaceans were not sampled. (*c*) Ekdale *et al.* [36] data with the addition of eight stem mysticete taxa. (*d*) Peredo *et al.* [26] data with the addition of the same eight stem mysticete taxa. In both datasets, stem mysticetes cluster with terrestrial artiodactyls and other non-mysticete groups rather than with extant Mysticeti. All panels share a common *y*-axis scale to facilitate direct comparison.

### 3.4. Finer taxonomic analysis

To identify which taxa drive the elevated mysticete signal, we subdivided the combined dataset into 14 subgroups (figure 3; see §2.2). A Kruskal–Wallis test revealed highly significant differences among subgroups (chi-squared = 63.2, d.f. = 13, *p* = 1.40 × 10^−8^). A one-way ANOVA corroborated this result (F = 7.65, *p* < 0.001).

Dunn's post hoc test with Bonferroni correction identified Balaenopteroidea as the primary driver of the mysticete signal. Balaenopteroidea was significantly different from Bovidae (*p* < 0.001), Cervidae (*p* < 0.001), Giraffidae (*p* = 0.007), Odontoceti (*p* = 0.001), stem Cetacea (*p* < 0.001), stem Mysticeti (*p* = 0.009) and Tragulidae (*p* < 0.001). In contrast, Balaenidae and Neobalaenidae was not significantly different from any artiodactyl family or from stem Mysticeti (all *p* > 0.05; figure 3). Stem Mysticeti was likewise not significantly different from any artiodactyl family, stem Cetacea or Odontoceti (all *p* > 0.05), sharing the ‘a’ designation with the majority of artiodactyl families in the compact letter display (figure 5).

**Figure 5.**
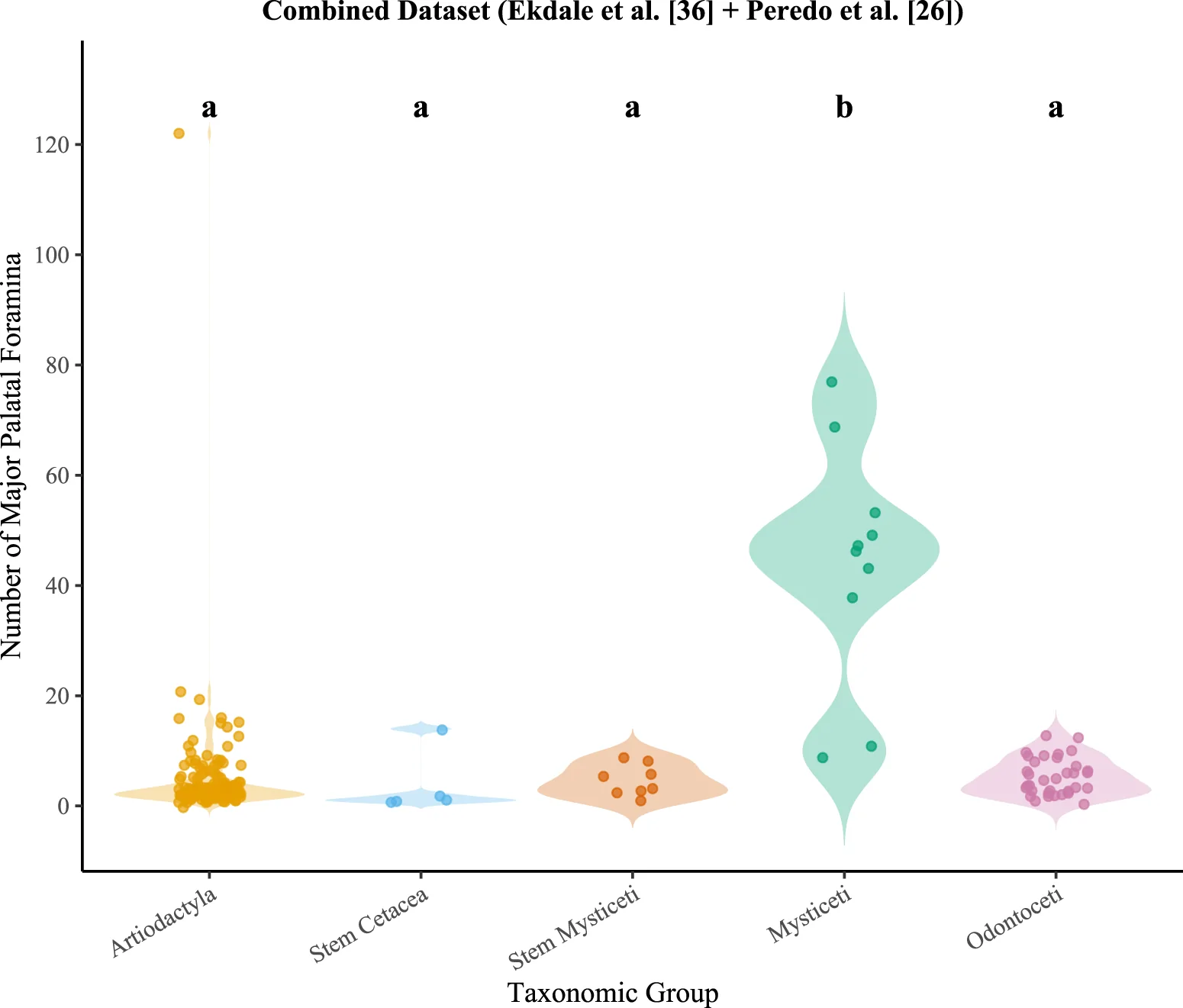
Violin plot of major palatal foramina counts across taxonomic groups in the combined dataset (Ekdale *et al.* [36] + Peredo *et al.* [26]), including eight stem mysticete taxa. Letters above each group indicate Tukey's HSD groupings from a one-way ANOVA (F = [X], *p* < [X]). Groups sharing the same letter are not significantly different. Extant Mysticeti (group ‘b’; *n* = 10, median = 46.5) is the only group significantly different from all others. Stem Mysticeti (group ‘a’; *n* = 8, median = 4) is statistically indistinguishable from Artiodactyla (*n* = 138, median = 3), stem Cetacea (*n* = 5, median = 1) and Odontoceti (*n* = 34, median = 5).

Notably, the elevated palatal foramina counts are not exclusive to Mysticeti. Hippopotamidae, the closest extant relatives of Cetacea, was significantly different from Bovidae (*p* = 0.018), Cervidae (*p* = 0.035), Giraffidae (*p* = 0.046), Odontoceti (*p* = 0.043), stem Cetacea (*p* = 0.002) and Tragulidae (*p* = 0.002). Hippopotamidae and Balaenopteroidea share the same significance grouping (‘c’) and are not significantly different from each other (*p* > 0.05; figure 3). The pattern observed in the five-group analysis is therefore not a distinction between Mysticeti and Artiodactyla, but rather a shared elevation in palatal foramina counts in Hippopotamidae and Balaenopteroidea relative to all other groups. Tayassuidae was also significantly different from Bovidae (*p* = 0.047), stem Cetacea (*p* = 0.006) and Tragulidae (*p* = 0.008).

## 4. Discussion

### 4.1. Stem mysticetes do not have superlative palatal foramina

The central question motivating the use of lateral palatal foramina as osteological correlates for baleen is whether stem mysticetes exhibit foramina counts comparable to those of extant, baleen-bearing mysticetes. Our results demonstrate unambiguously that they do not. Across all analyses—whether using the Peredo *et al.* [26] dataset, the Ekdale *et al.* [36] dataset, or the combined dataset—stem mysticetes are statistically indistinguishable from terrestrial artiodactyls, stem cetaceans and odontocetes in the number of major palatal foramina (figures 3–5). The highest count among stem mysticetes, *C. havensteini* at 9 foramina, falls well within the range of terrestrial artiodactyls and below the minimum observed in extant mysticetes.

Ekdale *et al.* [36] argued that extant mysticetes have significantly more palatal foramina than terrestrial artiodactyls. On this basis, they concluded that the presence of lateral palatal foramina is sufficient for inferring baleen in fossil taxa. However, their analysis included no fossil taxa (figure 4). When stem mysticetes are added to their dataset, the taxa for which baleen are inferred cluster with the groups that lack baleen, not with the groups that possess it (figure 4*c*). This result holds regardless of counting methodology, and it holds when the two datasets are merged under conditions that favour the Ekdale *et al.* [36] interpretation (figure 5).

If lateral palatal foramina were reliable indicators of baleen, we would expect putative baleen-bearing stem mysticetes—such as *A. weltoni* and *C. havensteini*—to exhibit foramina counts approaching those of extant mysticetes. Instead, these taxa have counts of 8 and 9, respectively, compared with a median of 46.5 in extant mysticetes. The results of our integrated analysis show that stem mysticete foramina counts are more consistent with the condition observed in terrestrial artiodactyls (median = 3) and toothed whales (median = 5) than with baleen-bearing mysticetes (see below for more discussion about defining baleen-bearing mysticetes).

### 4.2. The rorqual signal and the hippo problem

Finer taxonomic comparisons reveal that the significant difference between Mysticeti and other groups in the five-group analysis is not a general mysticete feature. Rather, it is driven almost entirely by Balaenopteroidea (i.e. Balaenopteridae + Eschrichtiidae *sensu* [33]). Balaenidae and Neobalaenidae, which also bear baleen, do not differ significantly from any artiodactyl family, stem Mysticeti or Odontoceti (figure 5). If palatal foramina counts were a reliable indicator of baleen, all baleen-bearing mysticetes should exhibit elevated counts. They do not.

The finding that Hippopotamidae shares the same statistical grouping as Balaenopteroidea further undermines the link between palatal foramina and baleen. Hippopotamids are the closest living relatives of cetaceans [42] and lack baleen, yet they exhibit palatal foramina counts that are statistically indistinguishable from rorquals and significantly elevated relative to most other artiodactyl families (figure 3). This shared condition between hippopotamids and balaenopteroids suggests that their elevated palatal foramina counts reflect convergence in highly vascularized gingival or oral soft tissue, rather than baleen specifically. Living hippopotamids possess notably thick, highly vascularized oral mucosa [43], as do rorquals, which must support the rapid growth and turnover of baleen plates [44] as well as the mechanical stresses of lunge feeding [45].

The most parsimonious explanation for these results is that palatal foramina counts reflect the degree of vascularization of the overlying soft tissue (gingiva, oral mucosa or Zwischensubstanz) rather than the presence or absence of any particular integumentary structure. In taxa with highly vascularized oral tissue, such as hippopotamids and balaenopteroids, elevated foramina counts should be expected, based on our results.

### 4.3. Methodological subjectivity in counting palatal foramina

The discrepancies between Peredo *et al.* [26] and Ekdale *et al.* [36] in counting palatal foramina are striking but unsurprising. The eight specimens common to both studies exhibit count differences ranging from twofold to over tenfold (supplementary material, table S3). These discrepancies arise because the palatal surface of mammalian skulls is inherently porous, particularly in the alveolar region, where trabecular bone produces numerous superficial openings that may or may not represent true patent foramina with confluent internal canals [38–40]. Distinguishing true foramina from post-mortem and taphonomic damage, superficial porosity or nutrient foramina of the periosteum requires subjective judgement that varies between observers and between methodological frameworks.

This subjectivity is itself evidence that palatal foramina are a flawed osteological correlate. A valid correlate should be identifiable consistently and unambiguously across observers. The fact that two experienced research teams examining the same specimens arrived at counts differing by an order of magnitude (e.g. *E. robustus*: 5 versus 69) demonstrates that palatal foramina do not meet this standard. A structure that cannot be reliably counted cannot reliably indicate the presence or absence of any specific soft tissue.

Moreover, the alveolar region of the palate is known to be anatomically plastic in other mammals that have undergone tooth loss (e.g. myrmecophages). Evidence from the mandibles of edentulous mammals demonstrates that alveolar bone is extensively remodelled following the loss of functional dentition [46–48]. The same process probably affects the palatal surface, meaning that the number and morphology of palatal foramina in edentulous stem mysticetes may reflect post-tooth-loss remodelling rather than the presence of any novel integumentary structure.

### 4.4. Implications for inferring baleen in fossil mysticetes

In the Introduction, we posed two hypotheses: (1) that extant mysticetes have a superlative number of palatal foramina, and (2) that the palatal foramina of stem mysticetes imply the presence of baleen. Our results provide a nuanced answer to the first and a clear rejection of the second.

Hypothesis 1 is partially supported. Extant mysticetes as a group do exhibit significantly more major palatal foramina than other groups in the five-group analysis (figures 4 and 5). However, the finer taxonomic analysis demonstrates that this signal is driven by Balaenopteroidea and is shared with Hippopotamidae (figure 3). Balaenidae and Neobalaenidae do not exhibit elevated counts despite bearing baleen. It is also worth noting that balaenopteroids are substantially over-represented in museum collections relative to balaenids and neobalaenids. This problem is particularly exacerbated by a dearth of voucher specimens of neobalaenids (e.g. *Caperea*) in Northern Hemisphere collections. Therefore, balaenopteroids are substantially oversampled in both the Peredo *et al.* [26] and Ekdale *et al.* [36] datasets. This sampling bias inflates the apparent mysticete signal in the five-group analysis by weighting it towards the one mysticete subgroup with elevated counts. The superlative condition is therefore not a property of baleen-bearing whales generally, but of a subset of mysticetes that share it with a non-cetacean outgroup.

Hypothesis 2 is not supported by any analysis in this study. Stem mysticetes are statistically indistinguishable from terrestrial artiodactyls in every comparison we conducted: (i) using the Peredo *et al.* [26] data; (ii) using the Ekdale *et al.* [36] data; and (iii) using the combined dataset with both broad and finer taxonomic splits. The taxa most frequently cited as evidence for baleen in stem mysticetes—*Aetiocetus weltoni* (8 foramina) and *Aetiocetus cotylalveus* (1 foramen)—have counts that fall squarely within the range of terrestrial artiodactyls and well below the range of any extant mysticete. There is no quantitative basis for using palatal foramina to infer baleen in these or any other stem mysticete.

Given these results, phylogenetic bracketing [30] remains the most defensible framework for inferring baleen in the fossil record. Morphological and molecular evidence support a single origin of baleen within crown Mysticeti [27–29]. Baleen can therefore be confidently inferred for fossil taxa recovered within the crown group. For stem mysticetes outside the crown, the baseline assumption from the ancestral state should be that baleen was absent unless direct evidence, such as preserved baleen or unambiguous phylogenetic placement within the crown, indicates otherwise.

### 4.5. Toothless mysticetes: what should we call them?

As Peredo *et al.* [19] noted, tooth loss had long been equated with the origin of baleen. Thus, it has often been assumed that toothless mysticetes must have had baleen. In fact, this confusion was codified when Mitchell [49] coined the clade name Chaeomysticeti based on a definition that mixes taxa and traits: ‘I here name the Chaeomysticeti or true baleen whales [Gr. *chaios*, genuine, true, good] encompassing living Mysticeti and defined as cetaceans with baleen and without a functional dentition in the adult stage’ [49]. While this definition was clearly intended to include fossil mysticetes outside of crown group Mysticeti, he characterizes Chaeomysticeti as both possessing baleen and lacking a functional dentition as adults. The relationship between toothlessness and the presence of baleen holds for living mysticetes, but these two synapomorphies need not have evolved at the same time; Peredo *et al.* [18] argued that, based on the stem mysticete *Maiabalena nesbittae*, they are decoupled. Unfortunately, the intent of Mitchell’s [49] concept of Chaeomysticeti is operationally limited without a phylogenetic framework (figure 6).

**Figure 6.**
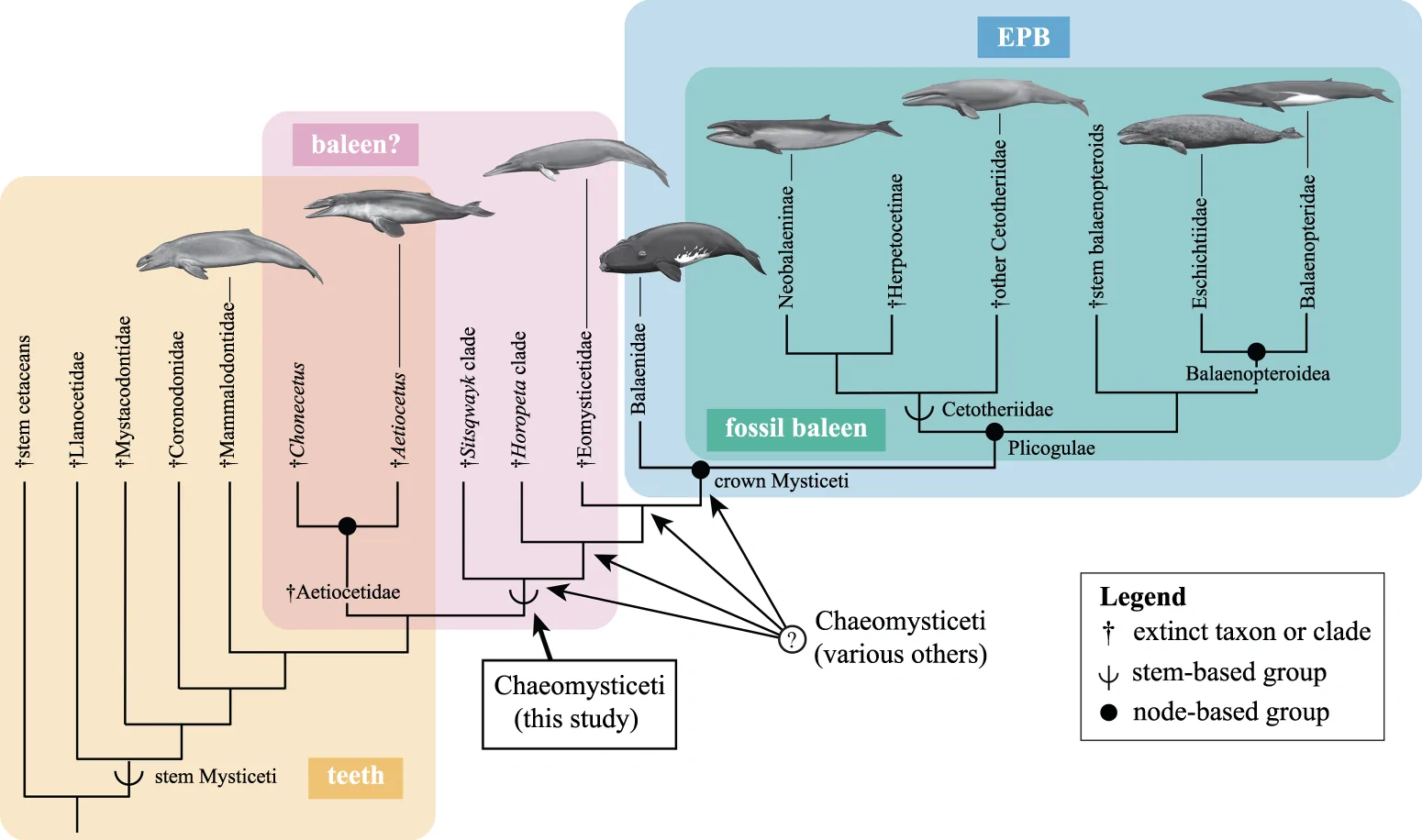
Simplified phylogeny of Mysticeti (based on [24,25,46,50]) showing alternative definitions of Chaeomysticeti relative to the presence of soft tissue traits. Crown Mysticeti, supported by extant phylogenetic bracketing (EPB, blue), supports the presence of baleen in all crown taxa; direct evidence of fossil baleen (green) has been demonstrated in Plicogulae (see text and [19–22]). Adult dentition (i.e. teeth in yellow) are documented in all stem cetaceans, early diverging stem mysticetes and Aetiocetidae; it is unclear if some chaeomysticetes possessed dentition. There is no direct evidence for baleen (red) in stem mysticetes belonging to the *Sitsqwayk* clade, the *Horopeta* clade, nor Eomysticetidae. Art by Carl Buell.

More recently, the equation of toothlessness and the presence of baleen was at least implied by Tsai *et al.* [50, figure 4] where they labelled the clade inclusive of *Sitsqwayk*, Eomysticetidae, *Horopeta* and crown Mysticeti as Chaeomysticeti. In their more detailed phylogeny, shown in their supplementary material, figure S2, *Sitsqwayk* is shown in a clade with *Maiabalaena* and *Tlaxcallicetus* (here called the *Sitsqwayk* clade), which is shown as the sister to the inclusive group defined by the *Horopeta* clade (*Whakakai*, *Taipahutea* and *Horopeta*) + Eomysticetidae + crown Mysticeti. Minimally, Tsai *et al.* [50] propose a phylogenetic basis for candidate baleen-bearing stem mysticetes.

If one assumes that baleen in living mysticetes had a single evolutionary origin, we can confidently infer the presence of baleen in all crown Mysticeti via extant phylogenetic bracketing. The one sure indication of the presence of baleen in a fossil mysticete taxon is the preservation of baleen itself. All known occurrences of fossilized baleen are associated with Plicogulae, which excludes Balaenidae and their fossil relatives [21].

Alternatively, it is entirely possible that baleen evolved multiple times in Mysticeti. First, tooth loss has evolved several times in odontocetes (the sister taxon to mysticetes) and sirenians as well [51,52]; instability in stem mysticete phylogeny makes it challenging to infer single or multiple origins of tooth loss in stem mysticetes. Even if baleen had a single origin, it may have been lost several times in the stem lineages of Mysticeti (i.e. in the *Sitsqwayk* clade, *Horopeta* clade or Eomysticetidae) in the same manner as ruminant headgear [53]. Given the diversity of keratinized palates in artiodactyls [43,54], the evolution of baleen in cetaceans may be more complex than suspected (regardless of tooth loss evolution). Such integumentary experimentations in the cranium (i.e. keratinized beaks) appear linked to tooth row reduction but not loss in dinosaurs [55].

Until we discover fossil baleen preserved in mysticetes outside of crown Mysticeti, there are three lineages that remain at odds with Mitchell’s [49] concept: the *Sitsqwayk* clade, Eomysticetidae and the *Horopeta* clade. Each clade has no functional adult dentition (although some taxa have indirect evidence of limited dentition), and they are phylogenetically outside of crown Mysticeti. Given the ambiguity of the presence or absence of baleen in these three lineages, a phylogenetic definition for Chaeomysticeti would be beneficial. We propose that Chaeomysticeti be defined as all mysticetes more closely related to crown Mysticeti than to Aetiocetidae. This definition encompasses Tsai *et al.*'s [50] node-based definition, while permitting topological changes and the addition of new lineages in the stem group. Also, this definition remains agnostic relative to the presence or absence of baleen in these stem lineages.

It remains entirely possible that fossil baleen or any keratinous structure could be discovered in a stem mysticete. Similarly, teeth are abundantly preserved in fossil mysticetes and it is possible that chaeomysticetes may have enamel-less vestigial teeth composed entirely of dentin (similar to fetal extant mysticetes [1,2,4]), as inferred by the pseudogenization of tooth-specific genes in extant cetaceans [56]. While we wait for future discoveries to point to a clear osteological correlate for baleen in chaeomysticetes, we urge consistency in phylogenetic nomenclature.

## 5. Conclusion

The data compiled by both Peredo *et al.* [26] and Ekdale *et al.* [36], when analysed together and supplemented with fossil taxa, converge on the same conclusion: lateral palatal foramina cannot be used to infer the presence of baleen in fossil whales. Stem mysticetes do not exhibit elevated palatal foramina counts relative to terrestrial artiodactyls, toothed whales or stem cetaceans. The elevated counts observed in extant mysticetes are driven by balaenopteroids and shared with hippopotamids, indicating that palatal foramina track soft tissue vascularization rather than the presence of baleen. Phylogenetic bracketing remains the most robust framework for inferring baleen in fossil cetaceans.

## Supplementary Material



## Data Availability

The data are available in the supplementary materials [57].
