# Supplementary material for: Lateral palatal foramina are ubiquitous across fossil whales and shared with hippos
Source: R Soc Open Sci. 2026 Oct 21;13(10):261050. doi: 10.1098/rsos.261050 (PMC13682885; doi:10.1098/rsos.261050)
Supplement: Supplementary file 1 [file rsos.261050.zip › rsos.261050/rsos261050_si_001.docx]

**Supplemental Tables for “Lateral palatal foramina are ubiquitous across fossil whales and shared with hippos”**

The following electronic supplemental materials include all the necessary data to reproduce our results.

### Table S1

**List of observed specimens and number of palatal foramina reported by Peredo et al. (2022).** Major palatal foramina were defined as those greater than 1 mm in diameter. See Methods for more details. Note that Peredo et al. (2022) used UMMVZ instead of UMMZ in Table S3 of their Supplementary Information.

| Institution | SpecNum | Genus | Species | Group | Total | Major |
| --- | --- | --- | --- | --- | --- | --- |
| UMMZ | 170469 | *Addax* | *nasomaculatus* | Artiodactyla | 5 | 1 |
| UMMZ | 124571 | *Aepyceros* | *melampus* | Artiodactyla | 25 | 8 |
| UMMZ | 163800 | *Alcephalus* | *beselaphus* | Artiodactyla | 7 | 2 |
| UMMZ | 163802 | *Alcephalus* | *beselaphus* | Artiodactyla | 9 | 3 |
| UMMZ | 75045 | *Alces* | *americanus* | Artiodactyla | 29 | 11 |
| UMMZ | 114769 | *Alces* | *americanus* | Artiodactyla | 10 | 6 |
| UMMZ | 97068 | *Ammotragus* | *lervia* | Artiodactyla | 3 | 1 |
| UMMZ | 65502 | *Antilocapra* | *americana* | Artiodactyla | 18 | 10 |
| UMMZ | 124085 | *Antilope* | *cervicapra* | Artiodactyla | 4 | 3 |
| UMMZ | 103248 | *Babyrousa* | *babyrousa* | Artiodactyla | 5 | 2 |
| UMMZ | 61294 | *Bison* | *bison* | Artiodactyla | 10 | 7 |
| UMMZ | 74590 | *Bison* | *bison* | Artiodactyla | 7 | 3 |
| UMMZ | 114844 | *Bos* | *frontalis* | Artiodactyla | 10 | 4 |
| UMMZ | 86878 | *Bos* | *taurus* | Artiodactyla | 17 | 8 |
| UMMZ | 167639 | *Boselaphus* | *tragocamelus* | Artiodactyla | 6 | 3 |
| UMMZ | 156568 | *Bubalus* | *bubalis* | Artiodactyla | 9 | 7 |
| UMMZ | 156569 | *Bubalus* | *bubalis* | Artiodactyla | 16 | 8 |
| UMMZ | 158958 | *Camelus* | *bactrionus* | Artiodactyla | 11 | 9 |
| UMMZ | 122700 | *Camelus* | *dromedarius* | Artiodactyla | 4 | 4 |
| UMMZ | 163707 | *Capra* | *hircus* | Artiodactyla | 13 | 3 |
| UMMZ | 125684 | *Capreolus* | *capreolus* | Artiodactyla | 7 | 2 |
| UMMZ | 124707 | *Catagonus* | *wagneri* | Artiodactyla | 27 | 14 |
| UMMZ | 125574 | *Catagonus* | *wagneri* | Artiodactyla | 22 | 16 |
| UMMZ | 178070 | *Cephalophus* | *zebra* | Artiodactyla | 10 | 2 |
| UMMZ | 59797 | *Cervus* | *elephas* | Artiodactyla | 7 | 4 |
| UMMZ | 124069 | *Connochaetes* | *taurinus* | Artiodactyla | 4 | 2 |
| UMMZ | 124075 | *Connochaetes* | *taurinus* | Artiodactyla | 2 | 1 |
| UMMZ | 124070 | *Damaliscus* | *korrigum* | Artiodactyla | 9 | 3 |
| UMMZ | 167702 | *Damaliscus* | *pygargus* | Artiodactyla | 13 | 6 |
| UMMZ | 103305 | *Eudorcas* | *thomsonii* | Artiodactyla | 6 | 2 |
| UMMZ | 124084 | *Eudorcas* | *thomsonii* | Artiodactyla | 2 | 1 |
| UMMZ | 102453 | *Gazella* | *dorcas* | Artiodactyla | 4 | 1 |
| UMMZ | 170490 | *Gazella* | *leptoceros* | Artiodactyla | 7 | 1 |
| UMMZ | 5488 | *Giraffa* | *camelopardalis* | Artiodactyla | 2 | 1 |
| UMMZ | 84041 | *Hippopotamus* | *amphibius* | Artiodactyla | 49 | 21 |
| UMMZ | 101782 | *Hippopotamus* | *amphibius* | Artiodactyla | 26 | 19 |
| UMMZ | 112380 | *Hippopotamus* | *amphibius* | Artiodactyla | 10 | 5 |
| UMMZ | 112381 | *Hippopotamus* | *amphibius* | Artiodactyla | 43 | 15 |
| UMMZ | 56528 | *Hydropotes* | *inermis* | Artiodactyla | 14 | 3 |
| UMMZ | 39518 | *Hyemosuchus* | *aquaticus* | Artiodactyla | 3 | 1 |
| UMMZ | 161034 | *Kobus* | *kob* | Artiodactyla | 1 | 1 |
| UMMZ | 92274 | *Lama* | *glama* | Artiodactyla | 6 | 3 |
| UMMZ | 103247 | *Lama* | *glama* | Artiodactyla | 2 | 2 |
| UMMZ | 124697 | *Mazama* | *americana* | Artiodactyla | 8 | 2 |
| UMMZ | 126854 | *Mazama* | *americana* | Artiodactyla | 4 | 1 |
| UMMZ | 80497 | *Mazama* | *gouazourbira* | Artiodactyla | 7 | 2 |
| UMMZ | 126127 | *Mazama* | *rufina* | Artiodactyla | 7 | 2 |
| UMMZ | 146496 | *Mazama* | *rufina* | Artiodactyla | 6 | 3 |
| UMMZ | 75243 | *Muntiacus* | *muntjac* | Artiodactyla | 2 | 1 |
| UMMZ | 173674 | *Muntiacus* | *reevesii* | Artiodactyla | 4 | 1 |
| UMMZ | 178188 | *Nanger* | *dama* | Artiodactyla | 5 | 3 |
| UMMZ | 124082 | *Nanger* | *granti* | Artiodactyla | 12 | 4 |
| UMMZ | 61156 | *Odocoileus* | *virginianus* | Artiodactyla | 5 | 2 |
| UMMZ | 61192 | *Odocoileus* | *virginianus* | Artiodactyla | 4 | 2 |
| UMMZ | 176280 | *Okapi* | *johnstoni* | Artiodactyla | 1 | 1 |
| UMMZ | 60546 | *Oreamnos* | *americanus* | Artiodactyla | 6 | 3 |
| UMMZ | 98132 | *Oreamnos* | *americanus* | Artiodactyla | 5 | 2 |
| UMMZ | 124068 | *Oryx* | *beisa* | Artiodactyla | 2 | 2 |
| UMMZ | 168361 | *Oryx* | *gazella* | Artiodactyla | 16 | 7 |
| UMMZ | 178562 | *Ourebia* | *orbi* | Artiodactyla | 4 | 2 |
| UMMZ | 116376 | *Ovibos* | *moschatus* | Artiodactyla | 8 | 2 |
| UMMZ | 98943 | *Ovis* | *ammon* | Artiodactyla | 4 | 1 |
| UMMZ | 97547 | *Ovis* | *aries* | Artiodactyla | 10 | 4 |
| UMMZ | 102446 | *Ovis* | *dalli* | Artiodactyla | 7 | 3 |
| UMMZ | 76668 | *Pecari* | *tajacu* | Artiodactyla | 31 | 10 |
| UMMZ | 76669 | *Pecari* | *tajacu* | Artiodactyla | 43 | 15 |
| UMMZ | 156549 | *Phacochoerus* | *aeithiopicus* | Artiodactyla | 10 | 2 |
| UMMZ | 156550 | *Phacochoerus* | *aeithiopicus* | Artiodactyla | 4 | 2 |
| UMMZ | 97462 | *Ranger* | *tarandus* | Artiodactyla | 12 | 4 |
| UMMZ | 97463 | *Ranger* | *tarandus* | Artiodactyla | 9 | 3 |
| UMMZ | 161756 | *Rusa* | *alfredi* | Artiodactyla | 6 | 3 |
| UMMZ | 159590 | *Rusa* | *mariana* | Artiodactyla | 12 | 4 |
| UMMZ | 157906 | *Sus* | *phillipinensis* | Artiodactyla | 2 | 1 |
| UMMZ | 157908 | *Sus* | *phillipinensis* | Artiodactyla | 6 | 2 |
| UMMZ | 158010 | *Sus* | *scrofa* | Artiodactyla | 8 | 7 |
| UMMZ | 176270 | *Taurotragus* | *derbianus* | Artiodactyla | 3 | 2 |
| UMMZ | 167403 | *Taurotragus* | *strepsiceros* | Artiodactyla | 19 | 8 |
| UMMZ | 76661 | *Tayassu* | *pecari* | Artiodactyla | 27 | 14 |
| UMMZ | 76665 | *Tayassu* | *pecari* | Artiodactyla | 22 | 6 |
| UMMZ | 178697 | *Tragulus* | *napu* | Artiodactyla | 2 | 1 |
| UMMZ | 86110 | *Tragulus* | *nigricans* | Artiodactyla | 4 | 2 |
| USNM PAL | 25210 | *Aetiocetus* | *cotylalveus* | Mysticeti | 1 | 1 |
| USNM | 593415 | *Balaenoptera* | *borealis* | Mysticeti | 25 | 11 |
| USNM PAL | 310633 | *Basilotritus* | *wardii* | Stem Cetacea | 2 | 2 |
| USNM | 550146 | *Caperea* | *marginata* | Mysticeti | 5 | 3 |
| USNM | 301637 | *Eubalaena* | *glacialis* | Mysticeti | 2 | 2 |
| USNM | 364973 | *Eschrichtius* | *robustus* | Mysticeti | 8 | 5 |
| USNM PAL | 546125 | *Isthminia* | *panamensis* | Odontoceti | 1 | 1 |
| USNM PAL | 256517 | *Simocetus* | *rayi* | Odontoceti | 3 | 3 |
| USNM | 504560 | *Tursiops* | *truncatus* | Odontoceti | 4 | 2 |
| USNM | 550969 | *Tursiops* | *truncatus* | Odontoceti | 4 | 2 |
| USNM PAL | 11049 | *Xenorophus* | *sloanii* | Odontoceti | 2 | 2 |
| USNM PAL | 11962 | *Zygorhiza* | *kochii* | Stem Cetacea | 1 | 1 |
| UMMP | 100139 | *Dorudon* | *atrox* | Stem Cetacea | 4 | 3 |
| UMMP | 93220 | *Dorudon* | *atrox* | Stem Cetacea | 2 | 2 |
| UMMP | 101222 | *Dorudon* | *atrox* | Stem Cetacea | 2 | 2 |

### Table S2

**List of observed specimens and number of palatal foramina reported by Ekdale et al. (2024).** MPF pal: palatal foramina in the palatines; MPF max: palatal foramina in the maxillae; MPF pre: palatal foramina in the premaxillae.

| Institution | SpecNum | Genus | Species | Family | Group | MPF-pal | MPF-max | MPF-pmx | Total |
| --- | --- | --- | --- | --- | --- | --- | --- | --- | --- |
| AMNH | 15898 | *Hippopotamus* | *amphibius* | Hippopotamidae | Artiodactyla | 5 | 77 | 40 | 122 |
| USNM | 61715 | *Balaenoptera* | *acutorostrata* | Balaenopteridae | Mysticeti | 2 | 75 | 0 | 77 |
| USNM | 364973 | *Eschrichtius* | *robustus* | Eschrichtiidae | Mysticeti | 12 | 57 | 0 | 69 |
| USNM | 16045 | *Balaenoptera* | *physalus* | Balaenopteridae | Mysticeti | 0 | 53 | 0 | 53 |
| USNM | 124326 | *Balaenoptera* | *musculus* | Balaenopteridae | Mysticeti | 0 | 49 | 0 | 49 |
| USNM | 593415 | *Balaenoptera* | *borealis* | Balaenopteridae | Mysticeti | 0 | 47 | 0 | 47 |
| USNM | 269982 | *Megaptera* | *novaeangliae* | Balaenopteridae | Mysticeti | 0 | 46 | 0 | 46 |
| USNM | 257513 | *Balaena* | *mysticetus* | Balaenidae | Mysticeti | 0 | 43 | 0 | 43 |
| USNM | 572922 | *Balaenoptera* | *ricei* | Balaenopteridae | Mysticeti | 1 | 37 | 0 | 38 |
| AMNH | 80232 | *Camelus* | *bactrianus* | Camelidae | Artiodactyla | 4 | 8 | 4 | 16 |
| AMNH | 14107 | *Camelus* | *dromedarius* | Camelidae | Artiodactyla | 2 | 10 | 1 | 13 |
| AMNH | 143520 | *Lagenorhynchus* | *albirostris* | Delphinidae | Odontoceti | 8 | 5 | 0 | 13 |
| AMNH | 24290 | *Giraffa* | *camelopardalis* | Giraffidae | Artiodactyla | 9 | 3 | 0 | 12 |
| AMNH | 34937 | *Delphinapterus* | *leucas* | Monodontidae | Odontoceti | 1 | 8 | 3 | 12 |
| AMNH | 248471 | *Catagonus* | *wagneri* | Tayassuidae | Artiodactyla | 2 | 9 | 0 | 11 |
| USNM | 550146 | *Caperea* | *marginata* | Neobalaenidae | Mysticeti | 0 | 11 | 0 | 11 |
| AMNH | 36595 | *Kogia* | *breviceps* | Kogiidae | Odontoceti | 4 | 6 | 0 | 10 |
| AMNH | 214946 | *Orcinus* | *orca* | Delphinidae | Odontoceti | 8 | 2 | 0 | 10 |
| USNM | 301637 | *Eubalaena* | *glacialis* | Balaenidae | Mysticeti | n/a | 9 | 0 | 9 |
| AMNH | 135639 | *Mesoplodon* | *europaeus* | Ziphiidae | Odontoceti | 0 | 9 | 0 | 9 |
| AMNH | 19315 | *Monodon* | *monoceros* | Monodontidae | Odontoceti | 3 | 6 | 0 | 9 |
| AMNH | 57330 | *Neophocaena* | *asiaeorientalis* | Phocoenidae | Odontoceti | 4 | 4 | 1 | 9 |
| AMNH | 30575 | *Pseudorca* | *crassidens* | Delphinidae | Odontoceti | 4 | 4 | 1 | 9 |
| AMNH | 98228 | *Bison* | *bison* | Bovidae | Artiodactyla | 6 | 2 | 0 | 8 |
| AMNH | 212982 | *Bos* | *taurus* | Bovidae | Artiodactyla | 4 | 4 | 0 | 8 |
| AMNH | 248739 | *Lama* | *pacos* | Camelidae | Artiodactyla | 2 | 4 | 2 | 8 |
| AMNH | 234938 | *Sus* | *scrofa* | Suidae | Artiodactyla | 6 | 0 | 2 | 8 |
| AMNH | 121098 | *Delphinus* | *delphis* | Delphinidae | Odontoceti | 7 | 1 | 0 | 8 |
| AMNH | 70573 | *Oreamnos* | *americanus* | Bovidae | Artiodactyla | 4 | 0 | 3 | 7 |
| AMNH | 15599 | *Ovibos* | *moschatus* | Bovidae | Artiodactyla | 5 | 2 | 0 | 7 |
| AMNH | 10200 | *Phocoena* | *phocoena* | Phocoenidae | Odontoceti | 2 | 3 | 2 | 7 |
| AMNH | 165102 | *Oryx* | *gazella* | Bovidae | Artiodactyla | 5 | 0 | 1 | 6 |
| AMNH | 54181 | *Phacochoerus* | *aethiopicus* | Suidae | Artiodactyla | 4 | 2 | 0 | 6 |
| AMNH | 94172 | *Tayassu* | *pecari* | Tayassuidae | Artiodactyla | 1 | 5 | 0 | 6 |
| AMNH | 143513 | *Leucopleurus* | *acutus* | Delphinidae | Odontoceti | 4 | 2 | 0 | 6 |
| AMNH | 205923 | *Pontoporia* | *blainvillei* | Pontoporiidae | Odontoceti | 2 | 4 | 0 | 6 |
| AMNH | 31418 | *Sagmatias* | *obliquidens* | Delphinidae | Odontoceti | 2 | 4 | 0 | 6 |
| AMNH | 237503 | *Stenella* | *coeruleoalba* | Delphinidae | Odontoceti | 4 | 2 | 0 | 6 |
| AMNH | 239115 | *Stenella* | *clymene* | Delphinidae | Odontoceti | 5 | 1 | 0 | 6 |
| AMNH | 152855 | *Babyrousa* | *babyrussa* | Suidae | Artiodactyla | 5 | 0 | 0 | 5 |
| UMMZ | 156568 | *Bubalus* | *bubalis* | Bovidae | Artiodactyla | 5 | 0 | n/a | 5 |
| UMMZ | 167702 | *Damaliscus* | *pygargus* | Bovidae | Artiodactyla | 2 | 0 | 3 | 5 |
| AMNH | 187204 | *Sus* | *philippensis* | Suidae | Artiodactyla | 5 | 0 | 0 | 5 |
| AMNH | 54313 | *Sylvicapra* | *grimmia* | Bovidae | Artiodactyla | 3 | 2 | 0 | 5 |
| AMNH | 35150 | *Sagmatias* | *cruciger* | Delphinidae | Odontoceti | 5 | 0 | 0 | 5 |
| AMNH | 11088 | *Stenella* | *attenuata* | Delphinidae | Odontoceti | 4 | 1 | 0 | 5 |
| AMNH | 54469 | *Bos* | *gaurus* | Bovidae | Artiodactyla | 4 | 0 | 0 | 4 |
| AMNH | 81796 | *Connochaetes* | *taurinus* | Bovidae | Artiodactyla | 2 | 0 | 2 | 4 |
| AMNH | 53405 | *Kobus* | *kob* | Bovidae | Artiodactyla | 4 | 0 | 0 | 4 |
| AMNH | 35235 | *Lama* | *glama* | Camelidae | Artiodactyla | 2 | 2 | 0 | 4 |
| AMNH | 57000 | *Ovis* | *ammon* | Bovidae | Artiodactyla | 4 | 0 | 0 | 4 |
| AMNH | 34278 | *Lissodelphis* | *borealis* | Delphinidae | Odontoceti | 2 | 2 | 0 | 4 |
| AMNH | 74485 | *Tursiops* | *truncatus* | Delphinidae | Odontoceti | 2 | 2 | 0 | 4 |
| AMNH | 100298 | *Bison* | *bonasus* | Bovidae | Artiodactyla | 2 | 1 | 0 | 3 |
| AMNH | 10266 | *Boselaphus* | *tragocamelus* | Bovidae | Artiodactyla | 3 | 0 | 0 | 3 |
| AMNH | 71740 | *Mazama* | *americana* | Cervidae | Artiodactyla | 3 | 0 | 0 | 3 |
| AMNH | 35964 | *Nanger* | *granti* | Bovidae | Artiodactyla | 3 | 0 | 0 | 3 |
| AMNH | 17972 | *Rangifer* | *tarandus* | Cervidae | Artiodactyla | 3 | 0 | 0 | 3 |
| AMNH | 187214 | *Rusa* | *marianna* | Cervidae | Artiodactyla | 2 | 1 | 0 | 3 |
| AMNH | 53528 | *Taurotragus* | *derbianus* | Bovidae | Artiodactyla | 3 | 0 | n/a | 3 |
| AMNH | 81160 | *Tragelaphus* | *strepsiceros* | Bovidae | Artiodactyla | 3 | 0 | 0 | 3 |
| AMNH | 235597 | *Globicephala* | *melas* | Delphinidae | Odontoceti | 3 | 0 | 0 | 3 |
| AMNH | 57333 | *Lipotes* | *vexillifer* | Lipotidae | Odontoceti | 0 | 3 | 0 | 3 |
| AMNH | 94169 | *Sotalia* | *fluviatilis* | Delphinidae | Odontoceti | 3 | 0 | 0 | 3 |
| AMNH | 120268 | *Sousa* | *teuszii* | Delphinidae | Odontoceti | 3 | 0 | 0 | 3 |
| AMNH | 239111 | *Stenella* | *frontalis* | Delphinidae | Odontoceti | 3 | 0 | 0 | 3 |
| AMNH | 37164 | *Steno* | *bredanensis* | Delphinidae | Odontoceti | 3 | 0 | 0 | 3 |
| AMNH | 113812 | *Addax* | *nasomaculatus* | Bovidae | Artiodactyla | 2 | 0 | 0 | 2 |
| UMMZ | 124571 | *Aepyceros* | *melampus* | Bovidae | Artiodactyla | 2 | 0 | 0 | 2 |
| AMNH | 34721 | *Alcelaphus* | *buselaphus* | Bovidae | Artiodactyla | 2 | 0 | 0 | 2 |
| AMNH | 122674 | *Alces* | *americanus* | Cervidae | Artiodactyla | 2 | 0 | 0 | 2 |
| AMNH | 35056 | *Ammotragus* | *lervia* | Bovidae | Artiodactyla | 2 | 0 | 0 | 2 |
| UMMZ | 65502 | *Antilocapra* | *americana* | Antilocapridae | Artiodactyla | 2 | 0 | n/a | 2 |
| AMNH | 19613 | *Antilope* | *cervicapra* | Bovidae | Artiodactyla | 2 | 0 | 0 | 2 |
| AMNH | 8354 | *Capra* | *hircus* | Bovidae | Artiodactyla | 2 | 0 | 0 | 2 |
| AMNH | 54921 | *Capreolus* | *capreolus* | Cervidae | Artiodactyla | 2 | 0 | 0 | 2 |
| AMNH | 89410 | *Cephalophus* | *niger* | Bovidae | Artiodactyla | 2 | 0 | 0 | 2 |
| AMNH | 146615 | *Cervus* | *elaphus* | Cervidae | Artiodactyla | 2 | 0 | 0 | 2 |
| AMNH | 212614 | *Damaliscus* | *lunatus* | Bovidae | Artiodactyla | 2 | 0 | 0 | 2 |
| AMNH | 23864 | *Dicotyles* | *tajacu* | Tayassuidae | Artiodactyla | 0 | 2 | 0 | 2 |
| AMNH | 187830 | *Eudorcas* | *thomsoni* | Bovidae | Artiodactyla | 2 | 0 | 0 | 2 |
| AMNH | 238347 | *Gazella* | *leptoceros* | Bovidae | Artiodactyla | 2 | 0 | 0 | 2 |
| AMNH | 82287 | *Gazella* | *dorcas* | Bovidae | Artiodactyla | 2 | 0 | 0 | 2 |
| AMNH | 53600 | *Hyemoschus* | *aquaticus* | Tragulidae | Artiodactyla | 2 | 0 | 0 | 2 |
| AMNH | 74877 | *Mazama* | *gouazoubira* | Cervidae | Artiodactyla | 2 | 0 | 0 | 2 |
| AMNH | 56996 | *Muntiacus* | *reevesi* | Cervidae | Artiodactyla | 2 | 0 | 0 | 2 |
| AMNH | 256675 | *Odocoileus* | *virginianus* | Cervidae | Artiodactyla | 2 | 0 | 0 | 2 |
| AMNH | 51219 | *Okapia* | *johnstoni* | Giraffidae | Artiodactyla | 2 | 0 | 0 | 2 |
| AMNH | 87211 | *Oryx* | *beisa* | Bovidae | Artiodactyla | 2 | 0 | 0 | 2 |
| AMNH | 118607 | *Ourebia* | *ourebi* | Bovidae | Artiodactyla | 2 | 0 | 0 | 2 |
| AMNH | 6239 | *Ovis* | *aries* | Bovidae | Artiodactyla | 2 | 0 | 0 | 2 |
| AMNH | 98960 | *Ovis* | *dalli* | Bovidae | Artiodactyla | 2 | 0 | 0 | 2 |
| AMNH | 107107 | *Tragulus* | *napu* | Tragulidae | Artiodactyla | 2 | 0 | 0 | 2 |
| AMNH | 203253 | *Tragulus* | *nigricans* | Tragulidae | Artiodactyla | 2 | 0 | 0 | 2 |
| AMNH | 245090 | *Grampus* | *griseus* | Delphinidae | Odontoceti | 2 | 0 | 0 | 2 |
| AMNH | 209105 | *Inia* | *geoffrensis* | Iniidae | Odontoceti | 1 | 1 | 0 | 2 |
| AMNH | 244110 | *Ziphius* | *cavirostris* | Ziphiidae | Odontoceti | 2 | 0 | 0 | 2 |
| AMNH | 36486 | *Hydropotes* | *inermis* | Cervidae | Artiodactyla | 1 | 0 | 0 | 1 |
| AMNH | 103980 | *Muntiacus* | *muntjak* | Cervidae | Artiodactyla | 0 | 0 | 0 | 0 |
| AMNH | 90802 | *Phocoenoides* | *dalli* | Phocoenidae | Odontoceti | 0 | 0 | 0 | 0 |

**Note:** *Megaptera novaeangliae* (USNM 269982) is listed under “Odontoceti” in the original Ekdale et al. (2024) dataset — this is a data entry error, as humpback whales are mysticetes.

### Table S3

**List of specimens observed and reported by both Peredo et al. (2022) and Ekdale et al. (2024).**

| Institution | Spec # | Genus | Species | Group | Peredo Major | Peredo Total | Ekdale MPF pal | Ekdale MPF max | Ekdale MPF pmx | Ekdale Total |
| --- | --- | --- | --- | --- | --- | --- | --- | --- | --- | --- |
| UMMZ | 124571 | *Aepyceros* | *melampus* | Artiodactyla | 8 | 25 | 2 | 0 | 0 | 2 |
| UMMZ | 156568 | *Bubalus* | *bubalis* | Artiodactyla | 7 | 9 | 5 | 0 | n/a | 5 |
| UMMZ | 167702 | *Damaliscus* | *pygargus* | Artiodactyla | 6 | 13 | 2 | 0 | 3 | 5 |
| UMMZ | 65502 | *Antilocapra* | *americana* | Artiodactyla | 10 | 18 | 2 | 0 | n/a | 2 |
| USNM | 550146 | *Caperea* | *marginata* | Mysticeti | 3 | 5 | 0 | 11 | 0 | 11 |
| USNM | 364973 | *Eschrichtius* | *robustus* | Mysticeti | 5 | 8 | 12 | 57 | 0 | 69 |
| USNM | 301637 | *Eubalaena* | *glacialis* | Mysticeti | 2 | 2 | n/a | 9 | 0 | 9 |
| USNM | 593415 | *Balaenoptera* | *borealis* | Mysticeti | 11 | 25 | 0 | 47 | 0 | 47 |
